# Coinfection of *Chlamydia* spp. and herpesvirus in juvenile farmed Siamese crocodiles (*Crocodylus siamensis*) in Thailand

**DOI:** 10.14202/vetworld.2021.1908-1914

**Published:** 2021-07-25

**Authors:** Weena Paungpin, Metawee Thongdee, Somjit Chaiwattanarungruengpaisan, Ladawan Sariya, Wanna Sirimanapong, Tanit Kasantikul, Rassameepen Phonarknguen, Poonnut Darakamas, Nlin Arya

**Affiliations:** 1 The Monitoring and Surveillance Center for Zoonotic Diseases in Wildlife and Exotic Animals, Faculty of Veterinary Science, Mahidol University, Nakhon Pathom, Thailand; 2 The Veterinary Aquatic Animal Research Health Care Unit, Department of Clinical Sciences and Public Health, Faculty of Veterinary Science, Mahidol University, Nakhon Pathom, Thailand; 3 Department of Pre-clinic and Applied animal Science, Faculty of Veterinary Science, Mahidol University, Nakhon Pathom, Thailand; 4 Veterinary Diagnostic Laboratory, Clemson Livestock Poultry Health, 500 Clemson Rd, Columbia, SC 29229, USA; 5 Prasu-Arthorn Animal Hospital, Faculty of Veterinary Science, Mahidol University, Nakhon Pathom, Thailand

**Keywords:** *Chlamydia* spp, herpesvirus, juvenile, pathology, phylogeny, Siamese crocodile

## Abstract

**Background and Aim:**

For a decade, chlamydial and herpesvirus infections have caused significant morbidity and mortality in farmed crocodiles. In September 2017, a total of 160 juvenile freshwater Siamese crocodiles (*Crocodylus siamensis*) with conjunctivitis/pharyngitis lesions were admitted at the Veterinary Aquatic Animal Research Health Care Unit, Faculty of Veterinary Science, Mahidol University. All crocodiles did not respond well to antibiotics or supportive treatments and died. This study aimed to detect and identify the causative agents associated with conjunctivitis/pharyngitis and fatal outcomes in juvenile farmed Siamese crocodiles.

**Materials and Methods:**

A total of 138 pharyngeal and conjunctival swabs and conjunctival scrapes were collected from live crocodiles. All swab and scrape samples were DNA-extracted and amplified by polymerase chain reaction (PCR) using *Chlamydiaceae-* and herpesvirus-specific primers. Tissue samples (brain, lung, liver, heart, spleen, and intestine) were collected from two representative postmortem animals. All tissue samples were processed for molecular and pathological analyses.

**Results:**

PCR examinations identified chlamydial and herpesvirus DNA in 92% (126/138) and 100% (138/138), respectively, of the tested swab and scrape samples. Of those positive samples, 79% (26/33), 67% (4/6), and 98% (97/99) of the pharyngeal swabs, conjunctival swabs, and conjunctival scrapes, respectively, were positive for both chlamydial and herpesvirus DNA. Histopathological examination indicated necrosis and mononuclear cell infiltration in the liver, kidney, and intestine of the affected animals. The intracytoplasmic accumulation of *Chlamydia* was randomly observed in the examined tissue sample. Moreover, the presence of chlamydial and herpesvirus DNA was also detected in the tissue samples, including the heart, intestine, brain, lung, liver, and spleen, of the affected animals by PCR. Phylogenetic analyses revealed that *Chlamydia* spp. detected in the juvenile Siamese crocodiles was notably different from other known species in the *Chlamydia* genus, while the herpesvirus detected in the crocodiles was closely related to crocodyline herpesvirus 1.

**Conclusion:**

Based on histopathological and molecular examinations, this report provided the first evidence of coinfection of *Chlamydia* spp. and crocodyline herpesvirus 1 in juvenile Siamese crocodiles in Thailand.

## Introduction

Farmed crocodiles, both freshwater (*Crocodylus siamensis* and *Crocodylus johnstoni*) and saltwater (*Crocodylus porosus*), suffered from *Chlamydia* spp. infection for a decade [1,2]. The apparent symptoms of chlamydial infections in crocodiles include conjunctivitis, pharyngitis, and hepatitis [1-3]. In Thailand, chlamydial infection has been recognized since 2012 [4], causing the major loss in hatching and juvenile Siamese crocodiles since then. To date, only three documents on chlamydial infection in crocodiles in Thailand were reported. The first report has been conducted during the chlamydiosis outbreak in Siamese crocodiles in 2012-2013 [2]. The presence of *Chlamydiaceae* has been investigated in 31 crocodile carcasses received from 14 farms across Central Thailand. The study demonstrated that the *Chlamydia* spp. detected in the tissue samples of Siamese crocodiles were genetically distinct from other known species in the family *Chlamydiaceae*. The second report revealed the histopathological and ultrastructural evidence of *Chlamydia* spp. that infected juvenile Siamese crocodiles, which died in the 2013 epizootic [4]. The study showed that chlamydial infection caused a systemic disease involving the liver, spleen, kidney, heart, and the whole of the respiratory tract in the infected crocodiles. Moreover, *Aeromonas sobria* was also isolated from the liver, spleen, and kidney of the examined crocodiles. Recently, Chaiwattanarungruengpaisan [5] has reported a novel species of freshwater crocodile’s *Chlamydia* spp. found in the south of Thailand.

Although *Chlamydia* was believed to be the cause of the pandemic syndrome in farmed crocodiles in Thailand, the study in Australia found a small association between the polymerase chain reaction (PCR) result and histological lesion [6,7]. Conversely, the association between herpesvirus and three disease syndromes, including conjunctivitis and/or pharyngitis, systemic lymphoid proliferation with non-suppurative encephalitis, and lymphonodular skin lesion, had been established. The novel crocodyline herpesviruses named crocodyline herpesviruses 1, 2, and 3 have been determined to be associated with the three emerging syndromes [8]. Interestingly, the combined presence of *Chlamydiaceae* and herpesvirus was observed in the crocodiles with conjunctivitis and/or pharyngitis syndrome [6]. However, the role of *Chlamydia* spp. and herpesvirus in the pathogenesis and epidemiology of the relevant syndromes requires further investigation. Nevertheless, herpesvirus infection in the freshwater crocodile in Thailand has not been reported.

The current estimates of the prevalence of chlamydial and herpesvirus infections in crocodiles are limited by the available input prevalence data and may not represent the actual prevalence in the general population. Additional relevant studies are required for better prevalence estimation at regional and global levels.

This study aimed to detect and identify the causative agents associated with conjunctivitis/pharyngitis and fatal outcomes in juvenile farmed Siamese crocodiles.

## Materials and Methods

### Ethical approval

All protocols involving animals used in this study have been reviewed and approved by the Institutional Animal Care and Use Committee at the Faculty of Veterinary Science, Mahidol University (protocol number: MUVS-2017-10-51).

### Study period and location

This study was conducted in September 2017 at Veterinary Aquatic Animal Research Health Care Unit, Faculty of Veterinary Science, Mahidol University, Nakhon Pathom, Thailand

### Crocodiles and sample collection

A total of 39 juvenile crocodiles were admitted at the Veterinary Aquatic Animal Research Health Care Unit, Faculty of Veterinary Science, Mahidol University, during the 1^st^ week of September 2017. The animals presented with moribund and weakness, exhibited poor growth, and had conjunctivitis/pharyngitis lesions. Another 121 crocodiles from the same farm were admitted during the following week. Pharyngeal and conjunctival swab samples were collected from the first batch of crocodiles presenting with clinical signs of pharyngitis and conjunctivitis, while conjunctival scrape samples were collected from the second batch of crocodiles. Tissue samples, which were composed of the brain, lung, liver, heart, spleen, and intestine, were collected from two representative postmortem animals. All types of collected tissues were kept at −20°C for molecular analysis. A part of individual tissue samples was fixed in 10% neutral buffered formalin at necropsy. The formalin-fixed samples were subsequently processed according to the standard procedure and stained using hematoxylin and eosin (H and E) and monoclonal antibody to *Chlamydia* lipopolysaccharide (sc-58108, Santa Cruz Biotechnology, USA) for further pathological analysis.

### Detection and identification of *Chlamydiaceae* and herpesvirus

Genomic DNA was extracted from all types of samples using the Genomic DNA Mini Kit (blood and cultured cell) (Geneaid, New Taipei City, Taiwan). The extracted DNA was finally suspended in 30 μL of Tris-EDTA buffer and stored at −20°C until assay. The diagnostic PCRs for *Chlamydiaceae* and herpesvirus detection were performed in all extracted DNA samples. A semi-nested PCR using primers specific to the *ompA* gene was carried out for *Chlamydiaceae* detection following a published method [9], and a nested PCR using primers targeting the conserved DNA-dependent DNA polymerase gene of herpesviruses was conducted for herpesvirus detection [10]. Positive control (recombinant plasmid DNAs harboring the fragment of either *ompA* gene or DNA polymerase gene), negative control (nuclease-free water), and extraction negative control (phosphate-buffered saline) were included in each run.

### Phylogenetic tree construction and analysis

Brain tissues that were PCR positive for both *Chlamydiaceae* and herpesvirus were pooled and used for further phylogenetic marker amplification. The amplified *ompA* of *Chlamydia* spp. and DNA-dependent DNA polymerase gene of herpesviruses from the diagnostic PCR were used as phylogenetic markers. The PCR products with predicted size were purified from agarose gel using GenepHlow™ Gel/PCR Kit (Geneaid, Taiwan) and directly sequenced using Macrogen (Korea).

The phylogeny of *Chlamydia* and herpesvirus has been constructed using the deduced amino acid sequence of the *omp* gene of *Chlamydia* and the DNA polymerase gene of the herpesvirus, respectively. The sequence detected in this study will then be compared with those sequences available in the GenBank database. The evolutionary history was inferred using the maximum likelihood method based on the Le_Gascuel_2008 model [11]. The initial tree(s) for the heuristic search were automatically obtained by applying the neighbor-joining and BioNJ algorithms to a matrix of pairwise distances estimated using a JTT model and then selecting the topology with the superior log-likelihood value. A discrete Gamma distribution was used to model the evolutionary rate differences among sites. Evolutionary analyses were conducted using MEGA 7.0.21 (https://www.megasoftware.net/) [12].

## Results

### Macro- and microscopic lesions

**Figure-1:**
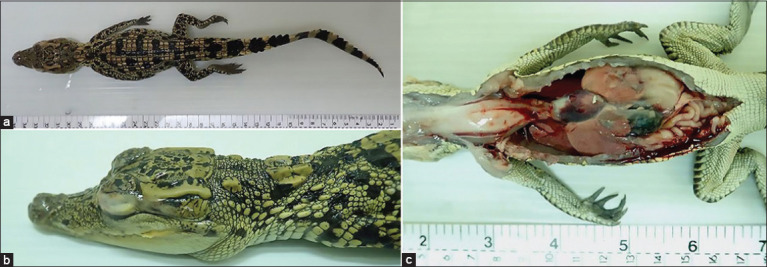
Macroscopic lesions. (a) Top view of the crocodile carcass showing poor nutrition status. (b) The eye was closed and covered with fibrinocaseous material. (c) Hydropericardium and pale brown liver with hundreds of pinpoint white foci.

The necropsy was conducted on two crocodiles. The animals were in poor nutritional status, and the coccygeal muscle of the proximal tail appeared to be small (Figure-1a). The conjunctivae of both crocodiles were bilaterally red and swollen and covered by fibrinocaseous material (Figure-1b). The oral mucosa and skin had multiple slightly raised yellow nodules, and the oral mucosa around the opening of the pharynx was also markedly red and swollen. In addition, both crocodiles had hydropericardium. All lungs appeared to be red and edematous, and the livers were observed to be pale and enlarged, with scattered white foci on the surface (Figure-1c). The kidneys were pale brown. The body fat was diffusely dull red in color.

**Figure-2:**
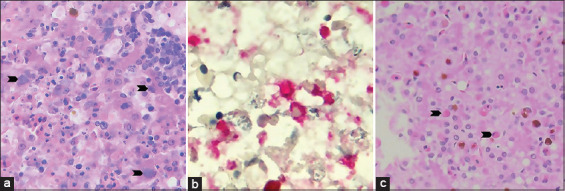
Histopathology of the liver sections. (a) Hematoxylin and eosin (H&E)-stained section showed the area of hepatocellular necrosis and cord disruption with associated infiltrates of mixed leukocytes. The intracytoplasmic clumps of granular basophilic coccoid bacteria were seen in the occasional hepatocytes and Kupffer cells (arrowhead). (b) The immunohistochemistry against monoclonal antibody to *Chlamydia* lipopolysaccharide on liver section (red color). (c) H&E-stained section showed rare hepatocytes containing large eosinophilic intranuclear inclusion bodies with marginating nuclear chromatin (arrowhead). Photos were taken using VetScan HDmicroscope (Abaxis, UK) at ×400 (Figure-2a and c) and 1000× (Figure-2b).

Histopathological examination revealed random areas of necrosis and infiltrates of lymphocytes, plasma cells, and heterophils. The intracytoplasmic accumulation of bacteria clumps that were positive from immunohistochemistry response to *Chlamydia* spp. was randomly observed (Figure-2a and b). Moreover, eosinophilic intranuclear inclusion bodies with marginating nuclear chromatin were found in rare hepatocytes (Figure-2c). Both animals were observed to have a marked lymphohistiocytic interstitial pneumonia. Mild to focally moderate lymphocytic perivascular cuffs were primarily scattered in the cerebral white matter. Similar mononuclear cell infiltrations were also observed in other viscera, including the kidneys and intestines.

### Detection and identification of *Chlamydiaceae* and herpesvirus

A total of 150 samples, consisting of 33 pharyngeal swabs, 6 conjunctival swabs, 99 conjunctival scrapes, and 12 tissues, were collected from the suspected crocodiles. The PCR detection of the samples collected at animal admission showed that 79% (26/33) and 67% of the pharyngeal and conjunctival swabs, respectively, were positive for both chlamydial and herpesvirus DNA, and the rest of those samples (seven pharyngeal swabs and two conjunctival swabs) were positive only for herpesvirus DNA (Table-1). The presence of both chlamydial and herpesvirus DNA in the crocodiles was detected to be as high as 98% (97/99) in the conjunctival scrape samples, which were collected a week after animal admission. Only 2% (2/99) of the scrape samples were found positive only for herpesvirus DNA (Table-1). Moreover, the PCR detection conducted in the tissue samples of the two representative animals showed that animal no. 1 had detectable herpesvirus DNA in all tissue samples (6/6, 100%), in which the heart and intestine samples were also found positive for chlamydial DNA. Conversely, animal no. 2 had detectable chlamydial DNA in all tissue samples (6/6, 100%), in which four samples, including the brain, lung, liver, and intestine, were also found positive for herpesvirus DNA (Table-2). Additional examination of the heart, lung, and liver samples of both animals found that they were PCR negative for West Nile virus, influenza A virus, and crocodile adenovirus.

**Table-1:** PCR results for *Chlamydiaceae* and herpesvirus detection in clinical samples from Siamese crocodiles.

Sample type	*Chlamydiaceae*/herpesvirus detection				Total

	+/+	+/-	-/+	-/-	
Pharyngeal swab	26	0	7	0	33
Conjunctival swab	4	0	2	0	6
Conjunctival scrape	97	0	2	0	99

PCR=Polymerase chain reaction

**Table-2:** PCR results for *Chlamydiaceae* and herpesvirus detection in necropsy tissue samples from Siamese crocodiles.

Animal no.	*Chlamydiaceae*/herpesvirus detection			

	+/+	+/-	-/+	-/-
1	Heart, intestine	None	Brain, lung, liver, spleen	None
2	Brain, lung, liver, intestine	Heart, spleen	None	None

PCR=Polymerase chain reaction

### Phylogenetic tree analyses

**Figure-3:**
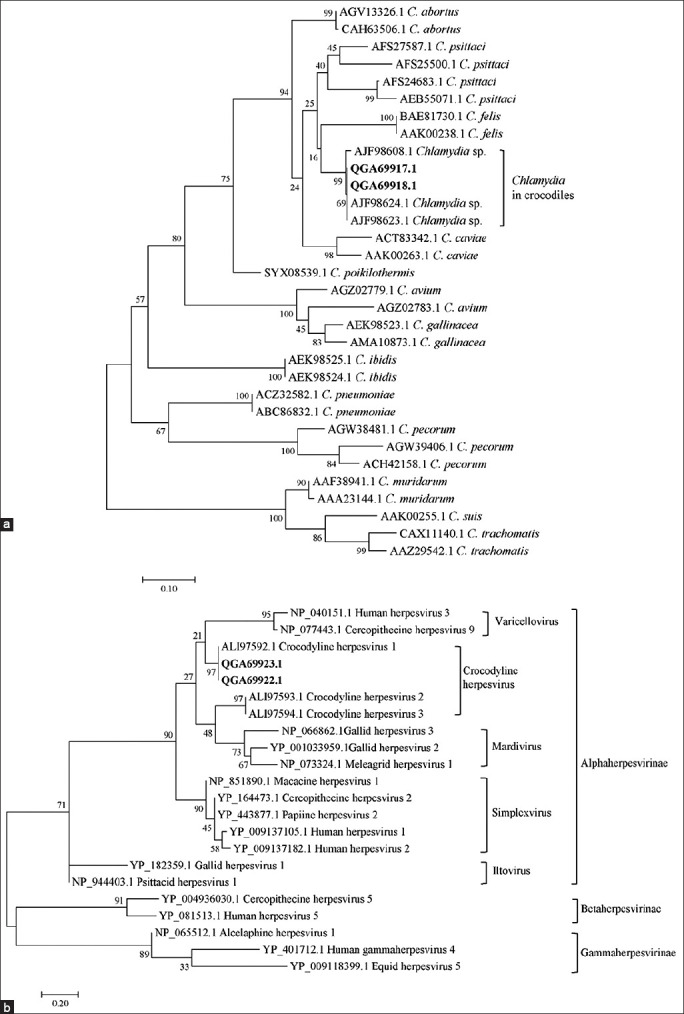
Phylogenetic trees constructed from the amino acid sequences of OMP protein (a) and DNA polymerase protein (b). The phylogenetic trees were constructed using the maximum likelihood method implemented in MEGA7.0.21. The percentage of tree in which the associated taxa clustered together is shown next to the branches. Each tree is drawn to scale, with branch lengths measured in the number of substitutions per site. The number shown in each species of *Chlamydia* and herpesvirus is the protein ID. Bold letters represented the sequences obtained from this study.

The OMP phylogeny showed that *Chlamydia* detected in Siamese crocodiles in this study were in the same group of crocodile *Chlamydia* as in the previous report [2] (Figure-3a). This crocodile *Chlamydia* was separated out of other known *Chlamydia*, providing species-specific information.

The phylogram of the herpesvirus DNA polymerase showed that the herpesvirus detected in the Siamese crocodiles was closely related to crocodyline herpesvirus 1 with a statistically bootstrap value of 97% (Figure-3b). Moreover, crocodyline herpesvirus 1 DNA polymerase sequences have a shorter evolutionary distance to the human alphaherpesvirus 3 (HHV-3) and Cercopithecine herpesvirus 9 (simian varicella virus [SVV]), which are members of the *Alphaherpesvirinae* in the genus *Varicellovirus*. HHV-3 is usually referred to as the varicella-zoster virus and the cause of chickenpox and herpes zoster in humans [13]. SVV causes a highly contagious infection of a variety of Old World non-human primates that can result in significant morbidity and mortality [14].

## Discussion

The emergence of chlamydial and herpesvirus infections causes significant morbidity and mortality in farmed crocodiles in several regions. Infections with *Chlamydia* spp. in crocodiles are typically recognized by ocular and respiratory symptoms [1,3].

The global prevalence of chlamydial infection in crocodile population was approximately 57.3% [15]. The prevalence rates were mostly determined in the sick crocodiles, although chlamydial infection has apparently existed in both normal and sick animals [7,15]. Based on the previous studies, *Chlamydia* positivity in crocodiles has ranged from 13.3% to 100% among examined carcasses and living animals [1-3,7,15,16]. In Thailand, nearly 75% (23/31) of crocodile carcasses derived from the chlamydiosis outbreak in 2012-2013 were positive for chlamydial infection [2], while infection with herpesvirus has less evidence. Few reports have been published, including reports in the farmed freshwater and saltwater crocodiles (*C*. *johnstoni* and *C*. *porosus*) in Australia [6,8,17] and the juvenile farmed American alligators (*Alligator mississippiensis*) [18].

Fulminant systemic disease with liver, spleen, kidney, heart, and respiratory tract involvement can occur particularly in infected hatchlings or juvenile crocodiles [4]. The recent study demonstrated that the syndrome of conjunctivitis and/or pharyngitis in crocodiles was associated with both *Chlamydia* spp. and herpesvirus infections [6]. In the present study, the macroscopic lesions of all crocodiles were similar to the findings of the previous studies including fibrinocaseous conjunctivitis, pharyngitis, hepatitis, and multiple slightly raised yellow nodules with erosion and necrosis at the oral mucosa and skin [1,3,4,6]. All examined crocodiles in this report showed PCR-positive result for herpesvirus, but not for *Chlamydia* spp. Microscopically, the lymphohistiocytic infiltration of internal tissues, including the lung and liver, which were found in the submitted animals, has been linked to herpesvirus infection in crocodiles [6,17,18]. Although eosinophilic intranuclear inclusion bodies that were observed in the hepatocytes indicated an early stage of infection [6,19,20], it could not confirm the association between these two infections. Nevertheless, both herpesvirus and *Chlamydia* spp. have obviously aggravated the illness of the crocodiles.

*Chlamydia* spp. detected in the juvenile Siamese crocodiles in this study was unique and differentiated from other known species in the *Chlamydia* genus based on the phylogenetic analysis. Our finding of a potentially novel species of *Chlamydia* spp. identified in the Siamese crocodile hosts was consistent with the previous reports [2,4]. The herpesvirus detected in the affected crocodiles was phylogenetically related to crocodyline herpesvirus 1. The crocodile-derived herpesviruses (crocodyline herpesvirus 1, 2, and 3) are clustered within the alphaherpesviruses [8]. Other members of alphaherpesvirus have varied mammalian origins, including Old World monkeys, avian species, and also humans [6]. The previous study in Australia reported that crocodyline herpesviruses 1 and 2 were only found in the saltwater crocodiles (*C. porosus*), while the CROCODYLINE herpesvirus 3 was only found in the freshwater crocodiles (*C. johnstoni*) [18]. Conversely, our study revealed the presence of crocodyline herpesvirus 1 in the freshwater Siamese crocodiles (*C. siamensis*) although the study lacked virus isolation from the crocodile samples.

## Conclusion

The results demonstrated that *Chlamydia* spp. and herpesvirus were molecularly detected and confirmed in the clinical and tissue samples of the infected juvenile farmed Siamese crocodiles in Thailand. The macro- and microscopic lesions in infected crocodiles supported this evidence. Phylogenetic analyses identified a potential novel species of *Chlamydia* and crocodyline herpesvirus 1 from the tissue samples of the fatal crocodiles. This report is the first to describe the coinfection of *Chlamydia* spp. and crocodyline herpesvirus 1 in Siamese crocodiles in Thailand. However, the pathogenesis is not well understood, and further investigation is required particularly on the association between the coinfection and the crocodile disease syndromes.

## Authors’ Contributions

WP, MT, LS, and NA: Designed the study and wrote the manuscript, WS, PD, SC, and NA: Collected and processed the samples, WP, MT, SC, and LS: Performed the molecular investigation and analysis, TK, RP, and NA: Performed the histological investigation and analysis. LS, TK, and NA: Interpreted and analyzed the data. All authors read and approved the final manuscript.
